# Enhancement of saquinavir absorption and accumulation through the formation of solid drug nanoparticles

**DOI:** 10.1186/s40360-018-0275-5

**Published:** 2018-12-04

**Authors:** Gabriel Kigen, Geoffrey Edwards

**Affiliations:** 1 Department of Pharmacology and Toxicology, Moi University School of Medicine, P.O. Box 4606, Eldoret, 30100 Kenya; 2 Department of Molecular and Clinical Pharmacology, University of Liverpool, Liverpool, L69 3GE UK

**Keywords:** Saquinavir, nanodispersion, solid drug nanoparticles, Caco-2 cell monolayers, transport, accumulation

## Abstract

**Background:**

Nanotechnology is now considered a promising drug delivery method for orally administered hydrophobic drugs to their sites of action. The effect of nanodispersion on cellular transport and accumulation of saquinavir (SQV) was investigated.

**Methods:**

The transport of five solid drug nanoparticle (SDN) SQV formulations along Caco-2 cell monolayers (CCM) was compared to that of standard SQV. The SDNs were prepared using SQV mesylate (20%), Pluronic F127 (10%) plus five other excipients (HPMC, PVP, PVA, Lecithin S75 and Span 80) in different proportions. Cellular accumulation in CEM parental and CEMVBL (P-gp overexpressing) cells was conducted to ascertain the effect of nanodispersion on P-gp mediated efflux of SQV. All SDN formulations were dissolved in water, whereas SQV in DMSO to improve solubility. Quantification was via HPLC.

**Results:**

From transport results, an SDN sample composed of SQV mesylate/Pluronic F127 plus HPMC (70%) and had a 24% increase in apparent absorption compared to standard SQV, largely driven by a 38% reduction in basolateral to apical permeation. Additionally, the formulation and two others (SQV mesylate/Pluronic F127 alone; and + HPMC (65%)/Lecithin [5%]) accumulated more significantly in CEM cells, suggesting enhanced delivery to these cells. Moreover, accumulation and transport of the three SDNs compared well to that of SQV despite being dissolved in water, suggestive of improved dissolution. The inclusion of PVA resulted in increased efflux.

**Conclusion:**

The use of HPMC and Pluronic F127 produced SQV SDNs with improved permeation in Caco-2 cells and improved accumulation in CEM cells, but negative effects with PVA.

## Background

Despite success of highly active antiretroviral therapy (HAART) in the management of HIV/AIDS, there are still several challenges including ability of the drugs to cross physiological barriers in order to reach HIV cellular reservoirs in sufficient quantities [1]. Although current therapy is able to lower the viral load below detectable limits, HIV virus continues to survive in anatomical areas with poor drug permeation (sanctuary sites) such as CD4+ macrophages leading to increased likelihood of development of resistance [1, 2]. Maximal suppression of the virus therefore requires drugs to adequately penetrate these reservoir sites, and nanotechnology is now considered as a potential drug delivery system [3]. The system has several advantages including increased solubility, improved delivery, bioavailability and simultaneous delivery of two or more drugs [4, 5]. It has also been demonstrated to circumvent the transporter mediated drug efflux, thus allowing the drugs to gain entry into cells [6, 7]. The potential for nanoparticles to deliver drugs to the CNS with minimal adverse effects has also been reported [8, 9].

Currently, there are two main nanotechnology techniques that are utilized in the improvement of physical properties of hydrophobic drugs with a view to enhancing their solubility [10, 11]. The first involves attachment or encapsulation of the drug to a vehicle (nano-carrier) such as polymeric materials and devices of nanometric size range [12]. Examples include liposomes, dendrimers, micelles and nanoemulsions [13, 14]. Drugs may also be linked to inorganic particles such as silica, iron, silver or gold [15]. The second involves manipulation of the drug to a particle dispersion (nanodispersion) which is then stabilized by a polymer and/or excipient to form solid drug nanoparticles (SDNs) [16]. Various polymer combinations and surfactants are used as excipients. Examples of the polymer combinations used include hydroxypropyl methylcellulose **(**HPMC), polyvinylpyrrolidone (PVP), polyvinyl alcohol (PVA), pluronic F68, pluronic F127, lecithin S75, Span 80, hydrolysed gelatin, kallicoat protect and polyethylene glycol 1k. The surfactants used include cremophor, solutol HS, Tween 20, Sisterna 16, hyamine, sodium alginate, and sodium caprylate [17]. The process involves the formation of oil-in-water emulsions (nanoemulsions) by dissolving hydrophobic drugs in a volatile water immiscible organic solvent and the water-soluble materials in water [18, 19]. Dry powder composites (nanocrystals) are then formed through freeze-drying the emulsions in order to remove the volatile/aqueous phases [16]. The resultant product composed of the drug dispersed throughout the water-soluble material is a stable, dry and highly porous compound [17, 19]. The method has been utilized to produce SDNs from several antiretroviral (ARV) drugs with better pharmacokinetic properties compared to the original drugs [16, 17, 20–22].

SQV is a potent ARV drug of the protease inhibitor (PI) class, but its use has been limited by its poor absorption with oral bioavailability of about 4% [23–25]. SQV mesylate was the first PI to be approved for the treatment of HIV/AIDS but was discontinued after a year owing to development of resistance which was largely attributed to its poor bioavailability [26]. It was replaced by a soft gelatin formulation with improved bioavailability in 1997, which was however withdrawn in 2006 due to adverse effects and replaced by SQV boosted with RTV [27, 28]. Like other PIs, SQV is a substrate of multidrug efflux transporters including P-gp which has been postulated to play a role in its therapeutic failures a result of reduced intracellular plasma concentrations due to increased flux [29–32]. For example, there is a 20-fold increase in SQV plasma concentrations; upon co-administration with RTV a potent P-gp Inhibitor; hence its use in boosting the drug [33]. Several attempts have therefore been made to improve its absorption through design of formulations that can increase its bioavailability, including SDNs [34–38]. Nanotechnology has been demonstrated to enhance delivery and bioavailability of SQV through increased solubility, improved transport and evasion of the P-gp-mediated drug efflux [38–40]. Previous studies using the nanocarrier approach have demonstrated enhanced bioavailability of the drug, including disposition into the CNS [41–45].

The main objective of this research work was to investigate the effects of nanodispersion on the cellular transport and accumulation of SQV. The permeation of standard SQV along the CCM was compared with that of five SDNs of the drug prepared using various excipients as described earlier [39]. The SDNs were dissolved in distilled water, while standard SQV was dissolved in DMSO to improve solubility. The transport along the CCM of each SDN was compared to that of standard SQV using a method validated earlier in our laboratory [46]. In order to ascertain the effects of nanodispersion on the cellular accumulation of SQV, a P-glycoprotein [P-gp] substrate, the accumulation of the SDNs in CEM parental and CEMVBL cells (which overexpress P-gp) was compared to that of standard SQV using a method also developed earlier in our laboratory [47].

## Methods

### Chemicals and reagents

Caco-2 cells were purchased from the European collection of cell cultures (ECACC No. 286010202), and the cells were counted using a Nucleo Counter (ChemoMetec, Lillerød, Denmark) cell counter. SQV was donated by Roche Pharmaceuticals (Welwyn Garden City, UK). The various SQV SDNs, were provided by IOTA NanoSolutions Ltd (MerseyBIO, Liverpool, UK). Clozapine (CLZ), Dulbecco's Modified Eagle Medium (DMEM), Roswell Park Memorial Institute medium (RPMI), Hanks' Balanced Salt solution (HBSS), Fetal bovine serum (FBS), Dimethyl sulfoxide (DMSO) and Trypsin-EDTA solution were purchased from Sigma Aldrich (Poole, UK). Acetonitrile (ACN) and methanol (MeOH) were purchased from VWR Laboratory Supplies (Poole, UK), whereas diethyl ether was purchased from Fisher Scientific (Loughborough, UK). All the other chemicals used were of analytical or HPLC grade. Deionized water used to prepare the solutions for mobile phase was purified in an Elga DV 25 pure lab option system (Egla, High Wycombe, Buckinghamshire, UK). T-lymphoblastoid cell lines, CEM and CEMVBL cells were gifts from Dr. R. Davey (University of Queensland, Brisbane, Australia).

### Equipment and chromatographic conditions

The HPLC system consisted of a Dionex (Dionex Softron GmbH, Germering, Germany) HPLC system with a P 680 pump, an ASI-100 automated sample injector and a UVD 1704 detector. A 250 μl injector with a 20 μl loop was used. Reversed-phase-liquid chromatography was carried out using a HyPurity TM 22105-154630 C18 analytical column, 5 μm x 4.6 mm x 150 mm (Thermo Electron Corporation, Runcorn, UK). A column guard (Thermo electron 60140-412) was used to protect the analytical column. The ultraviolet detector was set to monitor the 215-nm wavelength. The mobile phase for the analysis was composed of ammonium formate 20 mM (pH = 4.2), ACN and MeOH (57:38:5 v/v) and was prepared fresh for each assay. Separation was facilitated via isocratic elution at 1.5 ml/min flow rate and the run time was eight minutes for each separation. 20 μl of the formulations was injected for each run by means of an automated injector. The peak area ratios for the calibration curves and quantification were obtained and analysed using Chromelon software (version 6.5). CLZ was used as internal standard. A Millicell Electrical Resistance System [ERS] (Fisher Scientific, Leicestershire, UK) was used for measuring the transepithelial electric resistance (TEER). Transwells (six-well transwell polycarbonate tissue culture treated plates, 4.67 cm^2^, 24 mm diameter; 0.4 μm pore size) were purchased from Corning life Sciences (Costar, High Wycombe, Buckinghamshire; UK).

### Cell culture

The cells were cultured in DMEM and plated onto transwells at a density of 2 × 10^4^ cells/cm^2^ supplemented with 15% FBS; followed by incubation at 37 °C and 10% CO_2_ in a humidified chamber with media change after every 2-3 days as described earlier [46–48]. Transport experiments were conducted 15 to 20 days after seeding and TEER across the cell monolayers was monitored until they were considered appropriate for the experiment (typically when the TEER values were above 500 Ω). The range of the range of TEERs used in our experiments were between 658 and 819 Ω.

### Cumulative trans-epithelial transport of SQV and the SDN formulations

The compositions of the SQV SDNs (SQV O5, SQV O6, SQV O7, SQV O9 & SQV 13) are as shown in Table 1. Solutions of 10 mg/ml standard SQV and the five SQV SDNs were prepared by weighing and dissolving in the appropriate solvent. SQV was dissolved in DMSO, while the SDNs in distilled water.

**Table 1:** Properties (chemical compositions and sizes) of the SQV SDNs

Sample	% SQV mesylate	% HPMC	% PVP	% PVA	% Pluronic F 127	% Lecithin S 75	% Span 80	Initial sample PS/nm	Type
SQV 05	20	70			10			430	
SQV 06	20				10			117	M
SQV 07	20		70		10			100	M
SQV 09	20	65			10	5		196	
SQV 13	20			65	10		5	336	

*HPMC* Hydroxypropyl methyl cellulose, *PVP* Polyvinylpyrrolidone, *PVA* Polyvinyl alcohol, *M* Multimodal, *PS* Particle size

Each experiment was carried out using paired samples of standard SQV and an SQV SDN in order to maintain similar experimental conditions. TEER was measured prior to transport studies and each monolayer was washed using prewarmed HBSS and equilibrated with the transport medium (DMEM without FBS). The medium was aspirated from all apical (AP) and basolateral (BL) compartments of the transwells and replaced with 2 ml of the transport medium (DMEM alone) and equilibrated for 1 hr (37 °C, 10% CO_2_ incubator), after which the TEER was re-assessed. The medium was then removed from both compartments and replaced with an equal volume of pre-warmed medium containing 20 μg/ml of the sample under investigation (SQV or SQV SDN). For the AP→BL transport, 2 ml the medium containing the sample was placed on the apical chamber and 2 ml of the medium alone on the basolateral chamber, whereas 2 ml of medium containing the drug were placed on the basolateral and 2 ml of medium on the apical chamber for the BL→AP transport. Transport in each direction was conducted in quintuplicate. The transwell plates were then incubated (37 °C, 10% CO_2_ incubator) and 100 μl was sampled hourly from the AP and BL compartments over 4 hr and replaced by freshly pre-warmed transport medium. The sampled were quantified via an HPLC method described earlier using a HyPurity C_18_ column and ultraviolet detection set at a wavelength of 215 nm [46]. The mobile phase consisted of ammonium formate, acetonitrile and methanol (57:38:5 v/v), and separation was facilitated via isocratic elution at a flow rate of 1.5 ml/min with clozapine as internal standard. The integrity of the CCM for each experiment was monitored by measuring the TEER at the beginning (0 min) and the end of the experiment (240 min). The transepithelial passage was assayed from the AP to the BL side and in the opposite direction BL to AP of the CCM. The results were expressed as apparent permeability (*P*_app_, unit: cms^-1^), the amount of compound transported per second. Flux described the movement of a substance across the polarized Caco-2 monolayers either in absorptive (AP→BL) or secretary direction [BL→AP] [48]. *P*_app_ values were calculated for both AP to BL (*P*_app AP-BL_), and BL to AP (*P*_appBL-AP_) movement of the compound using the following equation:

$$ {P}_{\mathrm{app}}\left(\mathrm{cm}/\mathrm{s}\right)=\frac{\left(\mathrm{dQ}/\mathrm{dt}\right)}{\Big(1/\left({\mathrm{AC}}_{\mathrm{O}}\right)} $$

Where dQ/dt is the steady-state flux (μmol s^-1^), A the surface area of the filter (cm^2^) and C_O_ the initial concentration in the donor chamber (μM). The equation applies only to the sink conditions, whereby the receiver concentration should not exceed 10% of the donor concentration and was therefore applied only for the samples taken at 60 minutes [48]. The apparent absorption was calculated using the formula: (*P*_appAP-BL_/*P*_appBL-AP_).

### Cellular accumulation experiments

CEM parental and CEMVBL cells were cultured in 175 cm^2^ flasks containing RPMI (+ 10% FBS) in a humidified incubator (37 °C, 10% CO_2_) as previously described [47]. The cells were counted and media containing 10 million cells centrifuged (2000 × *g* for 5 min at 4 °C) and the pellet reconstituted to a cell count of one million cells per ml in 10 ml of fresh RPMI. This was followed by the addition of solutions of 100 μl of 1 mg/ml of SQV and the SQV SDNs to yield a drug concentration of 10 μg/ml in each flask. Each set of experiments consisted of seven flasks, one each for SQV and the five SQV SDNs. The seventh flask contained 100 μl of 1 mg/ml of SQV combined with excipients used for the preparation of the SQV 13 sample. The purpose of carrying out this experiment was to rule out the possibility that the powder may have been responsible for the apparently reduced transport of sample 13 based on the results from Caco-2 experiment. The cells were then incubated at 37 °C for 30 min in a shaking water bath and the resulting cell suspensions centrifuged (2000 × *g* for 5 min at 4°C). Aliquots of 100 μl of the supernatant fraction were then used to determine extracellular (EXT) concentration. The excess supernatant fraction was then removed and the resulting cell pellet washed three times in 10 ml HBSS and then centrifuged (2000 × *g* for 5 min). The resulting pellets were reconstituted in 100 μl of distilled water and used to determine intracellular (INT) concentrations. The formulations were then assayed by HPLC and data expressed as cellular accumulation ratio (CAR) as described earlier [47].

### Statistical analysis

Statistical analysis was performed using StatsDirect v.3 (UK). The results were presented as mean ± standard deviation (SD) of five individual experiments with 95% confidence intervals for differences between the means where appropriate. The assessment of normality was done using Shapiro-Wilk test and analysis performed using unpaired t-test as the data was found to be distributed normally. A two-tailed *p value of < 0.05 was accepted as being significant.

## Results

### Cumulative trans-cellular transport

**Fig. 1:**
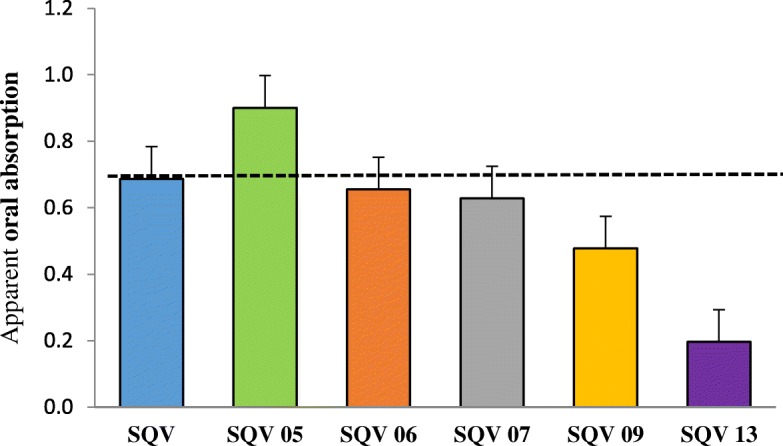
Apparent absorption of standard SQV and five SQV SDNs across the differentiated Caco-2 monolayers after 1 hour of incubation (n=5)

The results for the apparent absorption are as outlined in Fig. 1 and Table 2. The SQV 05 SDN formulation had a 23.7% (0.69 *vs* 0.90, *p* = 0.61) increase in the apparent absorption compared to standard SQV after 1 hour of incubation. Two other SDN samples (SQV 06 & 07) had a marginal decrease in the absorption compared to standard SQV, with that of SQV 13 being the most significant 0.69 at 71% (0.69 *vs* 0.20, *p* = 0.06) [Fig. 1]. It would therefore appear that the dissolution of these three samples improved, since they were dissolved in distilled water whereas standard SQV was dissolved in DMSO to increase solubility.

**Table 2:** Comparison of the apparent absorptions of five SQV SDNs to that of standard SQV across differentiated Caco-2 monolayers after 1 hour of incubation (n=5)

Sample	Apparent absorption *= P*app_AP-BL_*/ P*app_BL-AP_					
	SQV	SQV SDN 05	SQV SDN 06	SQV SDN 07	SQV SDN 09	SQV SDN 13
1	0.5	1.8	0.4	0.3	0.6	0.1
2	0.2	0.2	0.4	0.1	0.2	0.1
3	0.2	0.4	0.5	0.3	0.3	0.0
4	1.7	0.8	0.4	1.6	0.5	0.0
5	0.9	1.3	1.5	0.8	0.8	0.7
Mean	0.7	0.9	0.7	0.6	0.5	0.2
STDEV	0.6	0.7	0.5	0.6	0.2	0.3
		*p* = 0.61	*p* > 0.99	*p* = 0.88	*p* = 0.50	*p* = 0.06

**Fig. 2:**
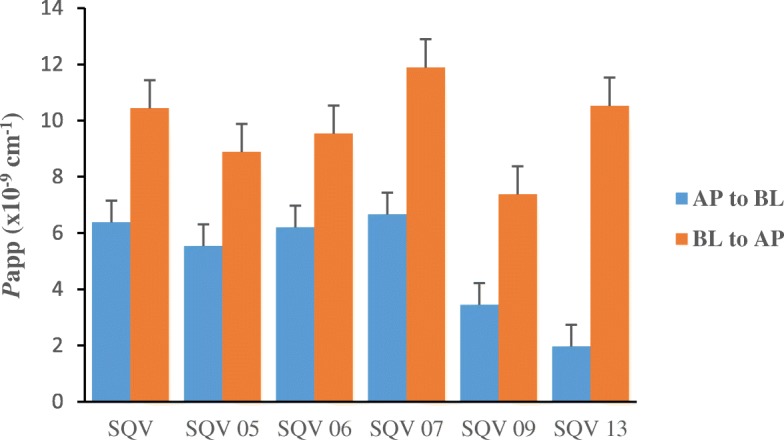
Apparent permeability of standard SQV and five SQV SDNs across the differentiated Caco-2 monolayers after 1 hour of incubation (n=5)

The apparent permeability coefficients are as shown in Fig. 2 and Table 3. The mean *P*app_BL-AP_ transport was higher than the *P*app_AP-BL_ across the spectrum as in the results from our previous study on SQV [46]. Additionally, the basolateral to apical permeation of SQV SDN 05 was higher than apical to basolateral permeation by 38% (8.9 *vs* 5.5) sample indicating that the increased apparent absorption was largely driven by a reduction in basolateral to apical permeation rather than by an enhancement in the apical to basolateral permeation (Fig. 2, Table 3).

**Table 3:** Comparison of the apparent permeabilities for the apical to basal and basolateral to apical transport of five SQV SDNs to that of standard SQV and across differentiated Caco-2 monolayers after 1 hour of incubation (*n* = 5)

a) Apical to basolateral								
	*P*app_A-B_ (x 10^-9^ cms^-1^)							
Sample	1	2	3	4	5	Mean	STDEV	*p* value
SQV	3.6	2.7	2.4	15	8.0	6.4	5.4	
SQV SDN 05	3.2	3.1	5.1	6.2	10	5.5	2.9	*p* = 0.77
SQV SDN 06	1.9	4.1	8.7	3.1	13	6.2	4.7	*p* = 0.96
SQV SDN 07	2.0	2.0	5.1	14	10	6.7	5.3	*p* = 0.94
SQV SDN 09	1.8	1.4	3.4	3.4	7.2	3.4	2.3	*p* = 0.30
SQV SDN 13	0.7	2.1	0.6	0.3	6.2	2.0	2.5	*p* = 0.06
b) Basolateral to apical								
	*P*app_B-A_ (x 10^-9^ cms^-1^)							
Sample	1	2	3	4	5	Mean	STDEV	*p* value
SQV	7.3	13	14	9.0	9.3	10	2.8	
SQV SDN 05	1.8	14	14	7.6	7.9	8.9	4.9	*p* = 0.56
SQV SDN 06	5.2	9.7	17	7.2	8.5	9.5	4.5	*p* = 0.71
SQV SDN 07	6.2	14	18	8.9	12	12	4.5	*p* = 0.56
SQV SDN 09	3.0	5.9	12	7.2	9.2	7.4	3.3	*p* = 0.15
SQV SDN 13	5.9	16	14	7.9	9.5	11	4.1	*p* = 0.97

### Accumulation experiments

**Fig. 3:**
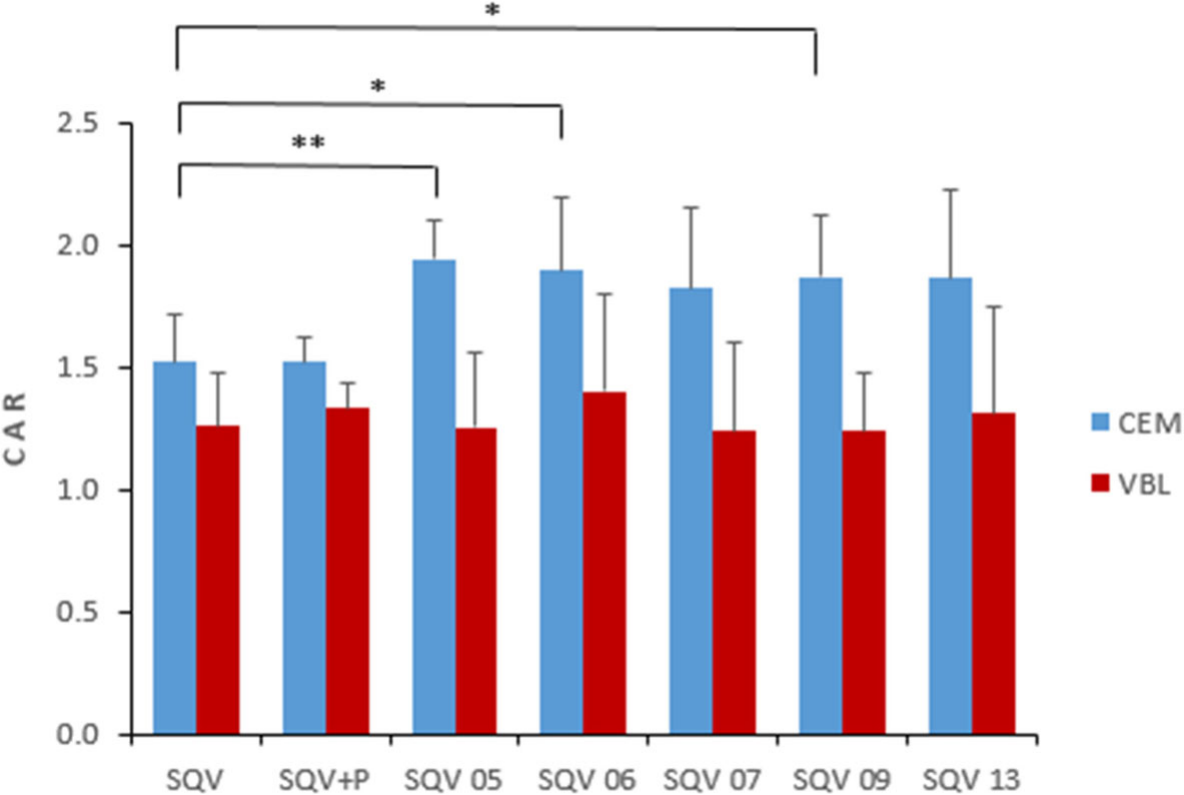
Cellular accumulation ratios [CAR] (Mean ± SD, n=5), * p <0.05, ** p <0.01

The intracellular accumulation results expressed as CAR are as shown in Fig. 3 and Tables 4. The intracellular accumulation of SQV in CEMVBL cells (which overexpress P-gp) was lower than CEM in all the formulations, suggesting that the formulations retained the P-gp substrate specificity as in our previous findings [47]. However, there was a significantly higher accumulation in CEM cells for three SDNs (SQV 05, 06 & 09) compared to that of standard SQV; 1.52 ± 0.19 versus 1.94 ± 0.16, *p* = 0.003 (SQV 05), vs 1.9 ± 0.29, *p* = 0.04 (SQV 06), and 1.87 ± 0.25, *p* = 0.04 (SQV 09) [Fig. 3, Table 4a]. These results suggest the possibility of enhanced delivery to these cells, despite the efflux. The excipients had no significant effect on the accumulation of SQV (Table 4a). Likewise, there was no significant difference in the accumulation of SQV and the SQV SDNs within the CEMVBL cells (Table 4b).

**Table 4:** Intracellular accumulation of SQV, SQV combined with excipients for SQV 13 and the SQV SDNs in CEM and CEMVBL cells

a) CEM cells								
	Cellular accumulation ration (CAR)							
Sample	1	2	3	4	5	Average	STDEV	*p* value
CEM SQV	1.5	1.3	1.8	1.6	1.4	1.5	0.2	
CEM SQV+P	1.5	1.6	1.5	1.7	1.4	1.5	0.1	*p* = 0.97
CEM SQV 05	2.2	1.8	1.8	1.9	2.0	1.9	0.2	*p* = 0.03
CEM SQV 06	2.2	1.5	2.1	2.0	1.7	1.9	0.3	*p* = 0.04
CEM SQV 07	2.1	1.4	1.8	2.2	1.6	1.8	0.3	*p* = 0.12
CEM SQV 09	1.9	1.6	1.9	2.3	1.7	1.9	0.3	*p* = 0.04
CEM SQV 13	1.8	1.6	1.5	2.1	2.4	1.9	0.4	*p* = 0.10
b) CEM VBL cells								
	Cellular accumulation ration (CAR)							
Sample	1	2	3	4	5	Average	STDEV	*p* value
VBL SQV	1.0	1.1	1.3	1.4	1.5	1.3	0.2	
VBL SQV+P	1.2	1.5	1.4	1.2	1.4	1.3	0.1	*p* = 0.48
VBL SQV 05	1.0	0.9	1.3	1.4	1.7	1.3	0.3	*p* = 0.99
VBL SQV 06	1.0	1.1	1.5	1.6	1.9	1.4	0.4	*p* = 0.49
VBL SQV 07	0.8	1.0	1.2	1.5	1.7	1.2	0.4	*p* = 0.93
VBL SQV 09	1.1	0.9	1.4	1.5	1.3	1.2	0.2	*p* = 0.92
VBL SQV 13	1.0	0.8	1.4	1.5	1.9	1.3	0.4	*p* = 0.80

P = Excipients for SQV 13 sample

## Discussion

The main aim of this study was to investigate the effect of nanodispersion on the permeation of SQV along the CCM, a measure of its absorption [48–51]. The transport of standard SQV was compared with that of five SQV SDNs. Cellular accumulation in CEM and CEMVBL cells was performed in order to ascertain the effect on nanodispersion on the P-gp mediated efflux of the drug. CCM are well suited for the evaluation of drug transport since they have similar morphological and functional properties to intestinal enterocytes. The permeability of drugs through CCM therefore correlates well with *in vivo* absorption in humans [50, 51]. In addition, they express a wide array of drug transporters (both efflux and influx) as well as metabolic enzymes, thus making them suitable for *in vitro* drug transport studies [50, 52, 53].

From our transport results, one SDN sample (SQV 05) showed a 24% improvement in apparent absorption compared to standard SQV. This was demonstrated to be largely driven by a reduction in basolateral to apical permeation since its basolateral to apical permeation was lower than the apical to basolateral by 38%. Intracellular accumulation indicated that SDN samples SQV 05, 06 & 09 accumulated more significantly in CEM cells, suggesting the possibility of enhanced delivery to these cells, despite the efflux. Additionally, their transport and accumulation compared well to that of standard SQV despite the fact that they were dissolved in water and SQV on DMSO to enhance its dissolution. This was suggestive of an improved dissolution. The excipients had no significant effect on the accumulation of SQV. These results suggest that SQV can potentially be manipulated to produce SDNs with improved absorption and accumulation by the use of suitable excipients. All the samples were composed of SQV mesylate (20%) and Pluronic F127 (10%), with SQV 06 having no extra excipient. SQV SDN 05 was composed of SQV mesylate (20%), HPMC (70%) and Pluronic F127 (10%). The use Polyvinyl alcohol (PVA) polymer and Span 80 (surfactant) however, produced an SDN (SQV 13) with a reduced transport (increased efflux), an undesired effect. The reduction in BL→AP permeability demonstrated by the SQV 05 sample may have resulted from several mechanisms including endocytosis of the intact drug, increased paracellular permeability or indirect mechanisms that enable enhanced permeation of the dissolved drug [54–56]. It may have also been due to a reduction in the a P-gp mediated efflux transport [29, 57]. SQV is a substrate of the multidrug efflux transporter P-gp, and its low oral bioavailability has been partly thought to be associated with the transporter [29, 32, 57, 58]. Previous studies have demonstrated increased *in vivo* bioavailability of SQV upon co-administration with of P-gp inhibitors [59]. It is noteworthy that the SQV SDNs were dissolved in water, whereas standard SQV was dissolved in DMSO in order to improve its solubility [39, 60–63]. This is an important observation as it means that the SDNs have potentially better dissolution than standard SQV, which has been a major drawback in its absorption [23–25, 64]. Increased dissolution/solubility would potentially improve the bioavailability of the drug, controlling for the efflux [5, 24, 65–67]. From the cellular accumulation results, intracellular accumulation was lower in CEMVBL than in CEM parental cells for all SDNs including the standard drug as in our previous results [47]. Nanodispersion did not therefore appear to significantly affect accumulation in CEMVBL cells in SDNs, suggesting that it did not significantly affect the P-gp mediated efflux transport. CEMVBL cells overexpress P-gp, and with SQV being its substrate, its net intracellular accumulation is reduced. Further *in vitro* assays may be conducted in order to corroborate this by the use of known P-gp inhibitors such as tariquidar or amiodarone [32, 47, 68, 69]. However, in CEM cells, three SDNs (SQV 05, 06 & 09) accumulated more significantly. This would perhaps suggest that at physiologically relevant P-gp density, there is enhanced delivery to these cells possibly via other transporters or through other mechanisms such as paracellular permeation [32, 54, 57]. It may have also resulted from an increase in solubility [5, 65–67].

Several studies on the effects of the ingredients on the cellular accumulation and transport of nanoparticles (NPs) in various cell lines have reported a number of influencing factors. The type of polymer and surfactant used impacts on the pharmacokinetics of a NP by altering its properties including composition, size/diameter, shape and surface chemistry including charge (zeta potential) [70–72]. F127 and PVA have been associated with small particle size while carboxymethylcellulose (Na-CMC) has been linked to NPs with larger sizes which tend to be eliminated faster from the body [72]. Increased accumulation of SDNs CEM cells has also been shown to be influenced by an increase in diameter, whereas increased zeta potential had a positive influence on CAR in Caco-2 cells, but a negative influence in HepG2 cells. In addition, PVA, Tween 80 and F 127 had a negative effect on accumulation in CEM cells, while F 68 had a positive influence on the permeability in Caco-2 cells [17]. From our results, HPMC and Pluronic F 127 had positive influence on accumulation, whereas PVA had a negative influence.

## Conclusions

The main finding from our study is that SQV can potentially be developed into SDNs with optimized pharmacological properties through nanodispersion. One SDN sample (SQV 05) showed a 24% improvement in the apparent absorption, which was largely driven by a by a 38% reduction in the basolateral to apical permeation. This sample was composed of SQV mesylate (20%), HPMC (70%) and Pluronic F127 (10%). In addition, three SDN formulations (SQV 05, 06 & 09) accumulated more significantly in CEM cells, suggesting that at physiologically relevant P-gp density, there is enhanced delivery to these cells possibly via other transporters, through increased solubility, or other mechanisms such as paracellular permeation. The transport and accumulation of these SQV SDNs also compared well to that of standard SQV despite being dissolved in water and SQV on DMSO to enhance solubility. This was suggestive of improved dissolution. However, the use Polyvinyl alcohol (PVA) and Span 80 produced an SDN (SQV 13) with increased efflux. Further research be may be conducted to ascertain *in vivo* effects*.*

## Abbreviations

ACN: acetonitrile
AIDS: Acquired Immune Deficiency Syndrome
AP: apical
ARV: antiretroviral
BL: basolateral
CAR: cellular accumulation ratio
CCM: Caco-2 cell monolayers
CNS: central nervous system
CTAB: cetrimonium bromide
DMEM: Dulbecco's Modified Eagle Medium
DMSO: Dimethyl sulfoxide
DOSS: dioctyl sodium sulfosuccinate
ER: efflux ratio
EXT: extracellular
FBS: fetal bovine serum
HBSS: Hanks' Balanced Salt Solution
HIV: Human Immunodeficiency Virus
HPLC: High Pressure Liquid Chromatography
HPMC: hydroxypropyl methylcellulose
INT: intracellular
MeOH: methanol
NP: Nanoparticles
*P*app: apparent permeability
P-gp: P-glycoprotein
PI: Protease inhibitor
PVA: polyvinyl alcohol
PVP: polyvinylpyrrolidone
RPMI: Roswell Park Memorial Institute medium
RTV: ritonavir
SDNs: solid drug nanoparticles
SDS: sodium dodecyl sulphate
SQV: saquinavir
TEER: transepithelial resistance
